# Integrated single-cell and experimental validation links effector chondrocyte-associated oxidative stress to EIF6-related p47phox–NOX2 signaling in osteoarthritis

**DOI:** 10.3389/fphys.2026.1882484

**Published:** 2026-08-07

**Authors:** Shuai Yuan, Huirun Chen, Jiaxue Wei, Jinxian Tan, Shuai Liu, Zhenyang Fu

**Affiliations:** 1 Department of Orthopedic Surgery, Central People’s Hospital of Zhanjiang, Zhanjiang, China; 2 School of Basic Medical Sciences, Southern Medical University, Guangzhou, China; 3 The First Clinical Medical School of Guangdong Pharmaceutical University, Guangdong Pharmaceutical University, Guangzhou, China

**Keywords:** effector chondrocytes, EIF6, osteoarthritis, oxidative stress, p47phox–NOX2 signaling, single-cell RNA sequencing

## Abstract

**Background:**

Oxidative stress is closely involved in osteoarthritis (OA), but the cell-type-specific oxidative stress landscape and related regulatory molecules in human cartilage remain incompletely defined. This study aimed to identify effector chondrocyte (EC)-associated oxidative stress features in OA and to prioritize candidate molecules linked to p47phox–NOX2-related ROS responses.

**Methods:**

Public human cartilage single-cell RNA sequencing datasets were integrated to construct an OA cartilage atlas. Oxidative stress activity was evaluated using multiple scoring methods, and high oxidative stress (HOS) cells were defined according to the top 10% of integrated oxidative stress scores. Cell-state and communication features were examined using CytoTRACE and CellChat. High-dimensional weighted gene co-expression network analysis, machine learning, protein–protein interaction network analysis, summary-data-based Mendelian randomization, and molecular docking were combined to prioritize candidate genes. EIF6 and YWHAB were examined in an inflammatory EC-like model, and EIF6-associated p47phox/TGF-β responses were further assessed in DMM-induced OA mouse tissues.

**Results:**

Single-cell analysis identified an EC population enriched in OA cartilage. ECs showed relatively high oxidative stress activity and represented the largest component of HOS cells. The proportion of ECs among HOS cells increased in OA samples, and HOS cells were concentrated near the EC-enriched region in the UMAP space. HOS/EC-enriched populations showed broad communication with other chondrocyte populations and were associated with inflammation, stress-related signaling, and extracellular matrix remodeling pathways. Integrated computational analyses prioritized EIF6 and YWHAB as candidate molecules associated with EC-related oxidative stress. In inflammatory EC-like cells, IL-1β and TNF-α increased EIF6 and YWHAB expression. EIF6 or YWHAB knockdown attenuated IL-1β-induced p47phox and NOX2 expression and reduced ROS-related fluorescence, with EIF6 knockdown showing a stronger effect. Co-immunoprecipitation suggested altered YWHAB–p47phox interaction under inflammatory stimulation. In DMM-induced OA tissues, EIF6 knockdown reduced p47phox upregulation, whereas EIF6 overexpression increased TGF-β staining, which was partly attenuated by p47phox knockdown.

**Conclusion:**

This study identifies EC-associated oxidative stress features in OA cartilage and prioritizes EIF6/YWHAB-associated p47phox–NOX2 signaling as a candidate axis linked to inflammatory ROS-related responses and tissue-level OA responses.

## 1 Introduction

Osteoarthritis (OA) is among the most common musculoskeletal disorders and a leading cause of pain, disability, and loss of mobility in older adults (Hunter and Bierma-Zeinstra, 2019). Although OA was once viewed largely as a disease of articular cartilage, it is now recognized as a whole-joint disorder involving cartilage, subchondral bone, synovium, meniscus, and periarticular tissues (Loeser et al., 2012). Current treatment remains focused mainly on symptom relief, whereas effective disease-modifying therapies are still limited. Defining the cell-state-specific molecular programs that shape OA pathology is therefore important for understanding disease mechanisms and identifying potential therapeutic targets.

Oxidative stress is one such program. Excess reactive oxygen species (ROS) can promote chondrocyte senescence, apoptosis, inflammatory signaling, and extracellular matrix degradation, all of which are relevant to OA progression (Zahan et al., 2020). NADPH oxidases are important sources of regulated ROS production, and experimental inhibition of these enzymes has been shown to attenuate OA development in preclinical models (Han et al., 2022). However, oxidative stress is unlikely to be distributed uniformly across the OA joint microenvironment. Which chondrocyte states show the strongest oxidative stress signatures, and which molecules are associated with these signatures, remain incompletely defined.

Single-cell RNA sequencing (scRNA-seq) has enabled the investigation of OA cartilage at cell-type and cell-state resolution. Previous scRNA-seq studies have revealed marked heterogeneity within human OA cartilage, including disease-associated chondrocyte states related to degeneration, inflammation, senescence, and matrix remodeling (Ji et al., 2019; Swahn et al., 2023). These findings argue against a uniform model of OA transcriptional change and instead suggest that specialized chondrocyte states may contribute to distinct components of disease biology. A cell-resolved view of oxidative stress in OA may therefore reveal pathogenic features that are obscured in bulk transcriptomic analyses.

Effector chondrocytes (ECs) represent a disease-associated chondrocyte state involved in inflammatory activation, stress responses, and extracellular matrix remodeling (Ji et al., 2019; Swahn et al., 2023). Because OA cartilage contains heterogeneous chondrocyte populations and is accompanied by inflammatory activation and osteochondral remodeling (Loeser et al., 2012; Scanzello and Goldring, 2012), EC-associated transcriptional programs may capture chondrocyte-state changes linked to oxidative stress, inflammation, and matrix turnover.

Here, we integrated human cartilage scRNA-seq datasets to evaluate oxidative stress activity across annotated chondrocyte populations. We identified an annotated EC population with prominent oxidative stress activity and found that ECs constituted a major component of high oxidative stress (HOS) cells. We then combined cell–cell communication analysis, high-dimensional weighted gene co-expression network analysis, machine learning, protein–protein interaction network analysis, summary-data-based Mendelian randomization, and molecular docking to prioritize candidate molecules associated with EC-related oxidative stress. Finally, an inflammatory EC-like model was used to evaluate EIF6/YWHAB-associated p47phox–NOX2 responses, and DMM-induced OA mouse tissues were used to assess EIF6-associated p47phox/TGF-β tissue responses.

## 2 Materials and methods

### 2.1 Study design and data sources

**Figure 1:**
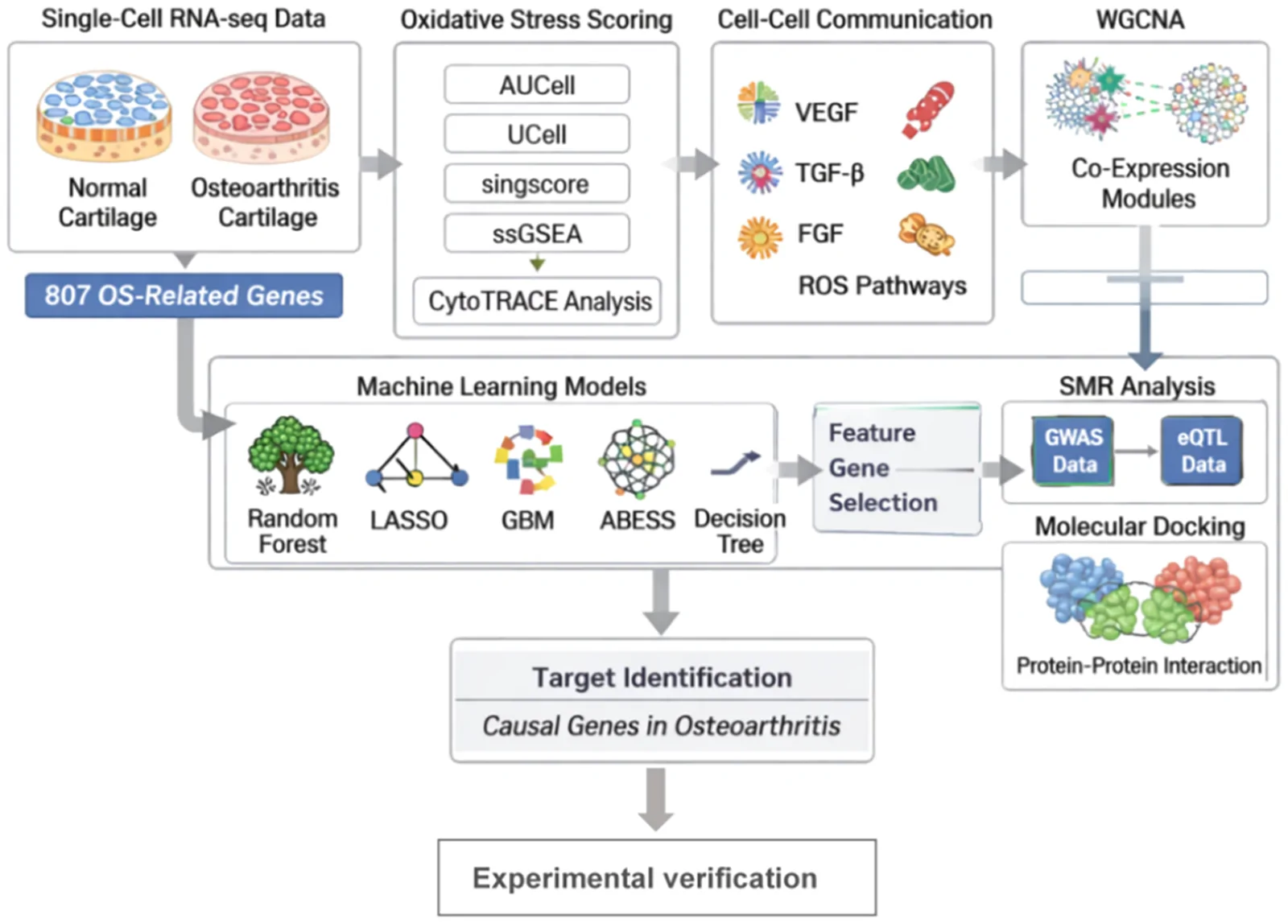
Integrated workflow for identifying effector chondrocyte-associated oxidative stress molecules in osteoarthritis. Public OA-related scRNA-seq datasets were integrated to generate a human cartilage atlas. Oxidative stress activity was quantified using multiple scoring methods, and HOS cells were then defined. EC-enriched oxidative stress features were examined by cell composition, UMAP distribution, and cell–cell communication analyses. hdWGCNA, machine learning, PPI network analysis, SMR, and molecular docking were integrated to prioritize candidate molecules. An inflammatory effector chondrocyte-like model and DMM-induced OA tissues were then used for validation.

The overall workflow is shown in **Figure 1**. OA-related human cartilage single-cell RNA-seq datasets, including GSE220243 and GSE169454, were obtained from the GEO database (Fu et al., 2021a; Swahn et al., 2022). The integrated dataset included 18 cartilage samples, consisting of nine normal and nine OA samples. OA GWAS summary statistics were obtained from the GWAS Catalog dataset GCST005812, and GTEx v8 whole-blood eQTL data were used for SMR analysis (Zengini et al., 2018; Buniello et al., 2019; Consortium GT, 2020).

### 2.2 Single-cell RNA-seq analysis

Single-cell RNA-seq data were analyzed using R and Seurat (Stuart et al., 2019; Hao et al., 2024). Low-quality cells were removed according to standard quality-control metrics, including detected gene number, transcript counts, and mitochondrial gene percentage (Luecken and Theis, 2019). After normalization and highly variable gene selection, datasets were integrated using Harmony to reduce batch effects (Korsunsky et al., 2019). PCA, graph-based clustering, and UMAP visualization were then performed (McInnes et al., 2018; Stuart et al., 2019). Cell clusters were annotated according to representative marker genes and published cartilage cell markers, including effector chondrocytes (ECs), regulatory-like chondrocytes, homeostatic chondrocytes, progenitor chondrocytes, hypertrophic chondrocytes, pre-hypertrophic chondrocytes, and fibro-chondrocytes. ECs refer to effector chondrocytes throughout this study.

### 2.3 Oxidative stress scoring and HOS cell identification

Oxidative stress activity was evaluated using a predefined oxidative stress-related gene set (Liberzon et al., 2015). Multiple scoring methods, including AUCell, UCell, singscore, ssGSEA, AddModuleScore, and an integrated scoring approach, were applied to improve robustness (Barbie et al., 2009; Hänzelmann et al., 2013; Aibar et al., 2017; Foroutan et al., 2018; Stuart et al., 2019; Andreatta and Carmona, 2021). Cells ranking within the top 10% of integrated oxidative stress scores were defined as high oxidative stress (HOS) cells. The 90th-percentile cutoff was used to identify cells with the most prominent oxidative stress activity while avoiding the inclusion of cells with only intermediate scores. The cell-type composition and UMAP distribution of HOS cells were then analyzed, with emphasis on the annotated EC population.

### 2.4 Cell-state, communication, and candidate gene analyses

CytoTRACE was used to infer the transcriptional state of HOS cells (Gulati et al., 2020). Cell–cell communication was analyzed using CellChat to evaluate ligand–receptor interactions, interaction number and strength, outgoing and incoming signaling patterns, and enriched pathways (Jin et al., 2021). High-dimensional weighted gene co-expression network analysis (hdWGCNA) was performed to identify co-expression modules associated with cell-type features and oxidative stress activity (Langfelder and Horvath, 2008). Candidate genes were further prioritized using random forest, LASSO, decision tree, GBM, and ABESS algorithms (Breiman et al., 1984; Tibshirani, 1996; Breiman, 2001; Friedman, 2001; Zhu et al., 2022). Overlapping genes were used for PPI network construction using STRING and Cytoscape (Shannon, 2003; Szklarczyk et al., 2025). SMR analysis was performed by integrating OA GWAS summary statistics with GTEx whole-blood eQTL data (Zhu et al., 2016). Because whole-blood eQTL data were used, SMR results were interpreted as supportive genetic prioritization evidence rather than confirmatory causal evidence in joint tissues or ECs. Molecular docking was used as supportive structural prediction evidence (Trott et al., 2009).

### 2.5 EC-like model culture, inflammatory stimulation, and transfection

C28/I2 human chondrocyte cells were used to establish an inflammatory effector chondrocyte-like model. For consistency with the single-cell annotation, this *in vitro* model is referred to as an inflammatory EC-like model in the experimental validation sections. Cells were cultured in DMEM high-glucose medium supplemented with 10% fetal bovine serum and 1% penicillin–streptomycin at 37 °C with 5% CO_2_. To establish an inflammatory EC-like model, cells were treated with IL-1β or TNF-α at 10 ng/mL for 24 h (Gan et al., 2022).

For loss-of-function experiments, cells were transfected with siRNAs targeting EIF6, YWHAB, or p47phox/NCF1, or with negative control siRNA. For gain-of-function experiments, cells were transfected with an EIF6 overexpression vector or empty vector. Transfections were performed using Lipofectamine 3000 according to the manufacturer’s instructions. Detailed information on cell source, culture conditions, siRNAs, and vectors is provided in **Supplementary Table 1**.

### 2.6 EC-like model validation and gene manipulation efficiency

The identity of the EC-like model was examined by qPCR analysis of chondrocyte-associated markers and vascular-lineage markers. Chondrocyte-related markers included SOX9, ACAN, COL2A1, and COMP. The vascular-lineage markers included PECAM1/CD31, CDH5, VWF, and EMCN. Inflammatory EC-like activation after IL-1β stimulation was evaluated by detecting inflammatory and matrix-remodeling markers, including IL6, CXCL8, PTGS2, and MMP13. Representative EC-associated markers, including KCNMA1, FST, and FGF1, were also assessed to connect the *in vitro* model with the single-cell EC annotation.

The efficiency of EIF6 knockdown, YWHAB knockdown, EIF6 overexpression, and p47phox/NCF1 knockdown was confirmed by qPCR and/or western blotting before downstream analyses.

### 2.7 ROS assays, western blotting, and co-immunoprecipitation

Intracellular ROS-related fluorescence was assessed using DCFH-DA staining (Kalyanaraman et al., 2012). Fluorescence images were acquired under identical exposure settings, and fluorescence signal was quantified as integrated density per field (Schneider et al., 2012). DHE staining was used as an additional ROS-related fluorescence assay, and fluorescence intensity was quantified as integrated density per field.

For western blotting, total protein was extracted from EC-like cells or mouse joint tissues, separated by SDS-PAGE, transferred to PVDF membranes, and incubated with antibodies against EIF6, YWHAB, p47phox, NOX2, and β-actin (Laemmli, 1970; Towbin et al., 1979). For co-immunoprecipitation, EC-like cell lysates were immunoprecipitated with an anti-YWHAB antibody, followed by western blotting for p47phox and YWHAB. Co-immunoprecipitation results were labeled as IP: YWHAB; WB: p47phox and IP: YWHAB; WB: YWHAB. Normal IgG was used as a negative control, and input lysates were included as loading controls.

### 2.8 DMM-induced OA mouse model

Male C57BL/6J mice aged 10 weeks and weighing 20–25 g were used to establish the DMM-induced OA model (Glasson et al., 2007). All animal procedures were reviewed and approved by the Laboratory Animal Welfare Ethics Committee of Central People’s Hospital of Zhanjiang under approval number PJ[DW-2025021-01]. Briefly, mice were anesthetized with sodium pentobarbital at 50 mg/kg by intraperitoneal injection, and adequate anesthesia was confirmed by the absence of pedal withdrawal reflex. Local 1% lidocaine was applied to the joint surface during surgery. The medial meniscotibial ligament was transected to induce joint instability, whereas Sham mice underwent joint exposure without ligament transection. Postoperative analgesia was provided with ibuprofen in drinking water at 30 mg/kg/day for 3 days.

For EIF6 knockdown experiments, mice were assigned to Sham, DMM + siNC, and DMM + siEIF6 groups. For p47phox-related tissue-response experiments, mice were assigned to DMM + Vector, DMM + EIF6-OE, and DMM + EIF6-OE + si-p47phox groups. *In vivo* gene manipulation reagents were delivered by intra-articular injection into the operated knee according to the experimental protocol. The dose, vehicle, injection volume, injection frequency, and injection schedule are provided in **Supplementary Table 1**. Four weeks after surgery, mice were euthanized by gradual-fill CO_2_ inhalation at a displacement rate of approximately 30–70% of the chamber volume per minute, followed by cervical dislocation to confirm death. Knee joint tissues were then collected for analysis.

### 2.9 Immunostaining and statistical analysis

For immunofluorescence, treated EC-like cells were fixed, permeabilized, blocked, and incubated with primary antibodies, followed by fluorescent secondary antibodies and DAPI counterstaining. For immunohistochemistry, decalcified paraffin-embedded knee sections were stained with antibodies against EIF6, p47phox, or TGF-β, developed with DAB, and quantified using ImageJ (Schneider et al., 2012). IHC staining was also used to validate the efficiency of *in vivo* EIF6 knockdown, EIF6 overexpression, and p47phox knockdown in the corresponding DMM groups.

Data are presented as mean ± SEM. For two-group comparisons, an unpaired two-tailed Student’s t-test was used when data met parametric assumptions; otherwise, a nonparametric test was applied. For comparisons among three or more groups, one-way ANOVA followed by Tukey’s or Dunnett’s *post hoc* test was used as appropriate. P < 0.05 was considered statistically significant.

## 3 Results

### 3.1 EC-centered analytical workflow for identifying oxidative stress-associated molecules in OA

We used an analytical strategy focused on EC-associated oxidative stress features to examine oxidative stress-associated molecular changes in OA (**Figure 1**). Human cartilage scRNA-seq datasets were integrated and annotated at the single-cell level. Oxidative stress activity was scored across the atlas, followed by the definition of high oxidative stress (HOS) cells. Because the annotated EC population was increased in OA samples and represented a major component of HOS cells, subsequent analyses focused on EC-associated oxidative stress features. CellChat was used to examine signaling involving HOS/EC-enriched cells, and hdWGCNA, machine learning, PPI network analysis, SMR, and molecular docking were combined to prioritize candidate genes. EIF6/YWHAB-associated p47phox–NOX2 signaling was then evaluated in an inflammatory effector chondrocyte-like model and DMM-induced OA tissues.

### 3.2 Single-cell profiling identifies an effector chondrocyte population enriched in OA cartilage

**Figure 2:**
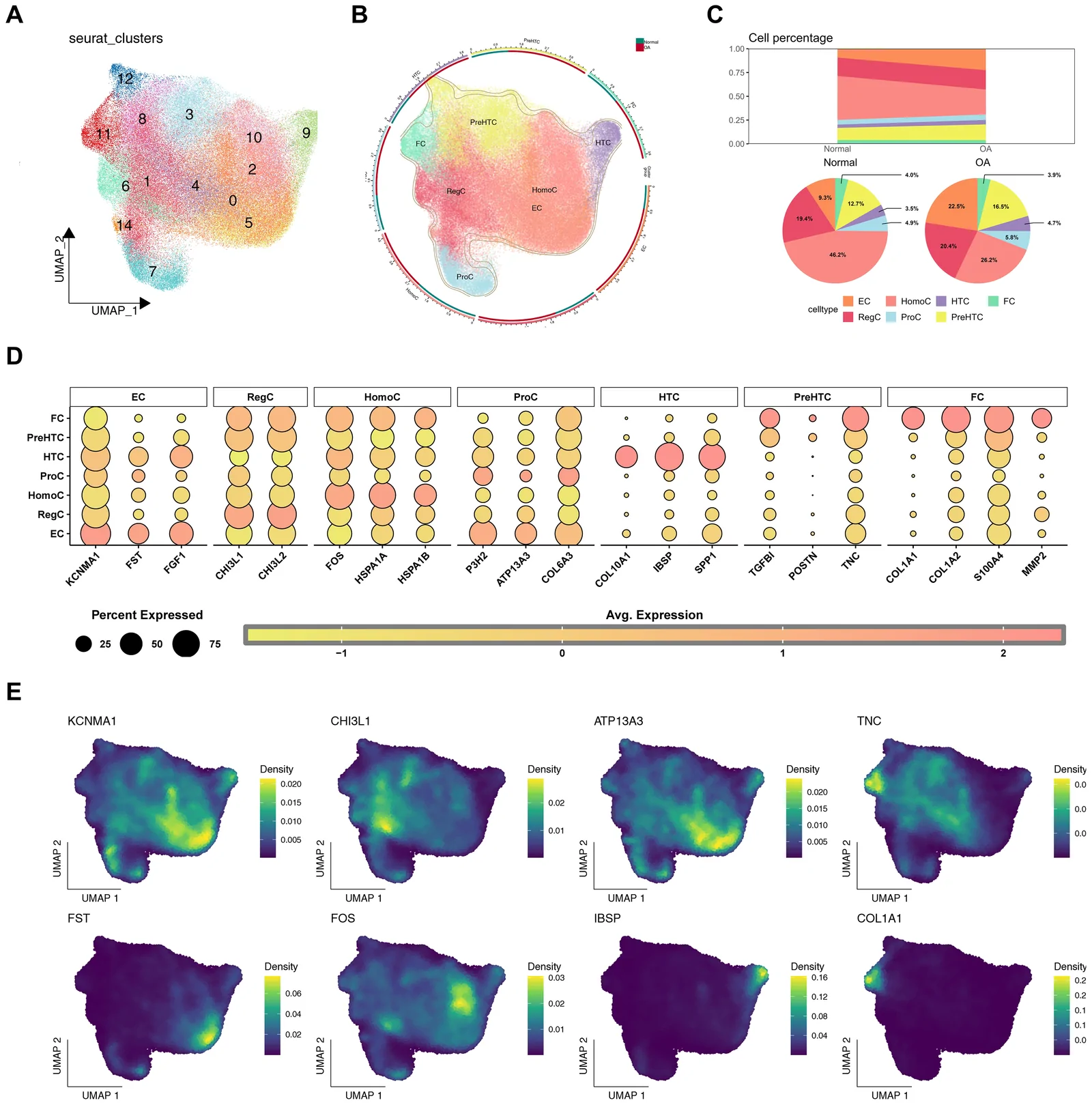
Single-cell atlas of human cartilage and annotation of chondrocyte subpopulations in OA. **(A)** UMAP visualization of Seurat clusters in the integrated scRNA-seq dataset. **(B)** Annotated UMAP visualization showing the distribution of chondrocyte subpopulations. **(C)** Cell-type proportions in normal and OA cartilage samples, with percentages labeled in the pie charts. **(D)** Dot plot showing representative marker genes for annotated chondrocyte subpopulations. **(E)** Density feature plots showing representative marker expression patterns. EC, effector chondrocytes; RegC, regulatory-like chondrocytes; HomoC, homeostatic chondrocytes; ProC, progenitor chondrocytes; HTC, hypertrophic chondrocytes; PreHTC, pre-hypertrophic chondrocytes; FC, fibro-chondrocytes.

After integration and clustering, cells formed multiple transcriptionally distinct groups in the UMAP space (**Figure 2A**). The annotated UMAP plot showed several chondrocyte subpopulations, including effector chondrocytes (ECs), regulatory-like chondrocytes (RegCs), homeostatic chondrocytes (HomoCs), progenitor chondrocytes (ProCs), hypertrophic chondrocytes (HTCs), pre-hypertrophic chondrocytes (PreHTCs), and fibro-chondrocytes (FCs) (**Figure 2B**).

Cell composition differed between normal and OA cartilage. The EC population was increased in OA samples, whereas the HomoC population was markedly reduced. Other populations showed comparatively modest compositional changes (**Figure 2C**). Marker expression supported the annotation of these chondrocyte subpopulations (**Figures 2D, E**). The EC annotation was based on effector chondrocyte-associated markers and published chondrocyte-subpopulation annotations. Together, these results established the cellular framework for subsequent analyses of oxidative stress associated with the EC subpopulation.

### 3.3 The annotated EC population exhibits prominent oxidative stress activity and represents a major component of HOS cells

**Figure 3:**
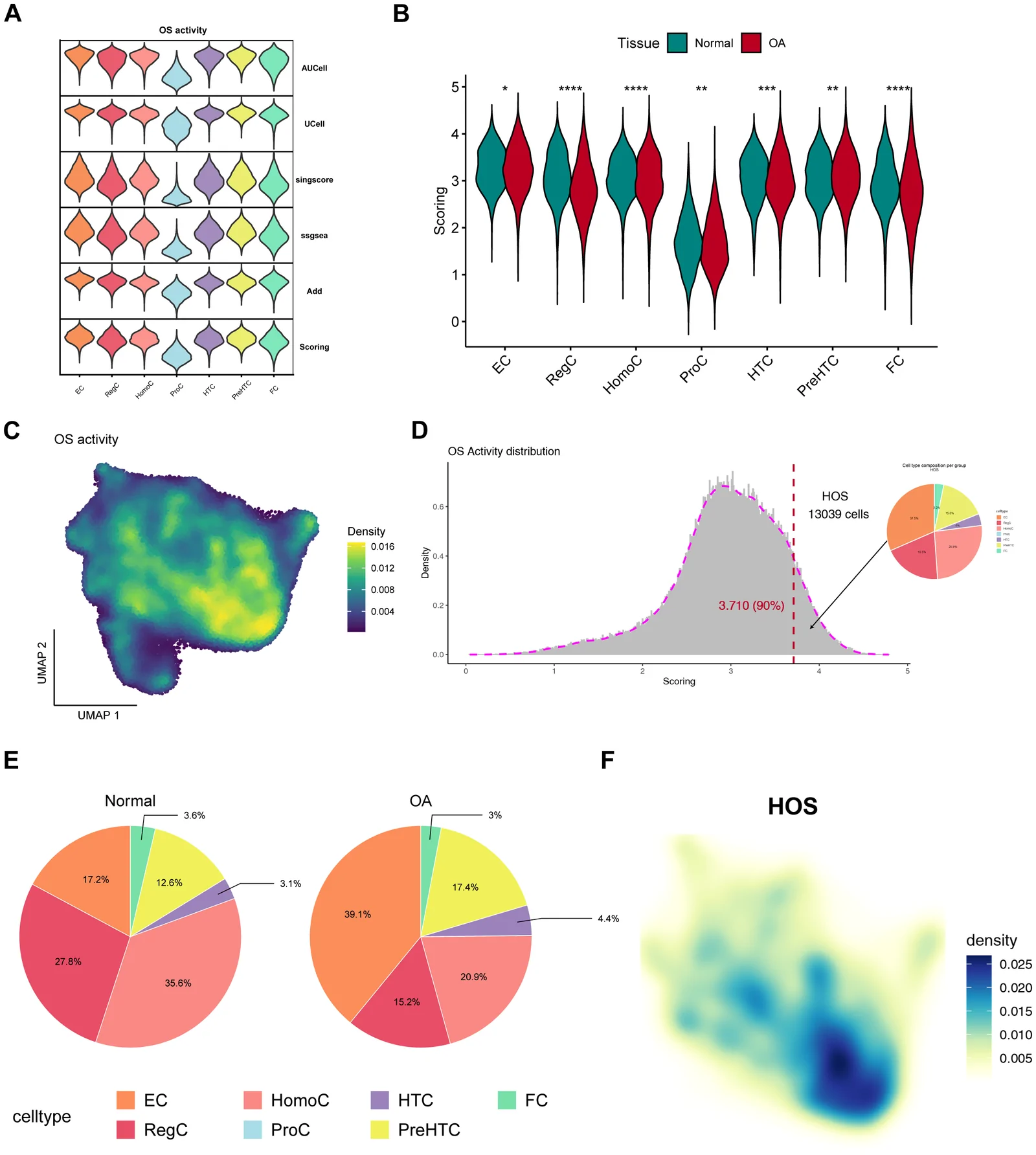
EC-associated oxidative stress activity and enrichment of HOS cells in OA cartilage. **(A)** Violin plots of oxidative stress scores across annotated cell populations. **(B)** Oxidative stress scores in normal and OA samples within each cell type. **(C)** UMAP visualization of integrated oxidative stress activity. **(D)** Distribution of integrated oxidative stress scores and the 90th-percentile threshold used to define HOS cells, together with the overall cell-type composition of HOS cells. **(E)** Cell-type composition of HOS cells in normal and OA samples. **(F)** UMAP density distribution of HOS cells. HOS, high oxidative stress; EC, effector chondrocytes. *P < 0.05; **P < 0.01; ***P < 0.001; ****P < 0.0001.

Oxidative stress scores varied substantially across annotated cell populations. The annotated EC population showed relatively high oxidative stress activity (**Figure 3A**), and oxidative stress scores showed cell-type-dependent differences between normal and OA cartilage. ECs exhibited increased oxidative stress scores in OA samples, whereas several other chondrocyte populations showed distinct or opposite shifts (**Figure 3B**). Scores generated by independent algorithms showed a consistent overall pattern, as illustrated by the dot plot, ridge plot, scoring distribution, and group-specific UMAP analyses (**Supplementary Figures 1A–E**).

Oxidative stress activity was spatially uneven across the atlas. Regions with higher scores overlapped with the EC-enriched area in the UMAP landscape (**Figure 3C**). Cells ranking within the top 10% of integrated oxidative stress scores were defined as HOS cells using the 90th-percentile cutoff (**Figure 3D**). Among HOS cells, the annotated EC population formed the largest component, accounting for approximately 31.5% of HOS cells (**Figure 3D**). When HOS cells were analyzed separately in normal and OA samples, the EC proportion increased from 17.2% in normal cartilage to 39.1% in OA cartilage (**Figure 3E**). HOS cells were also concentrated within or near the EC-enriched UMAP region (**Figure 3F**). Thus, the annotated EC population was both enriched in OA samples and strongly represented among cells with high oxidative stress activity. Although HOS cells were not restricted to ECs, the high-score region and HOS density distribution largely overlapped with the EC-annotated region. Therefore, subsequent analyses focused on the overall HOS population and interpreted these results in the context of EC-associated oxidative stress features.

### 3.4 HOS/EC-enriched cells show communication programs related to inflammatory, stress-associated, and matrix-remodeling pathways

**Figure 4:**
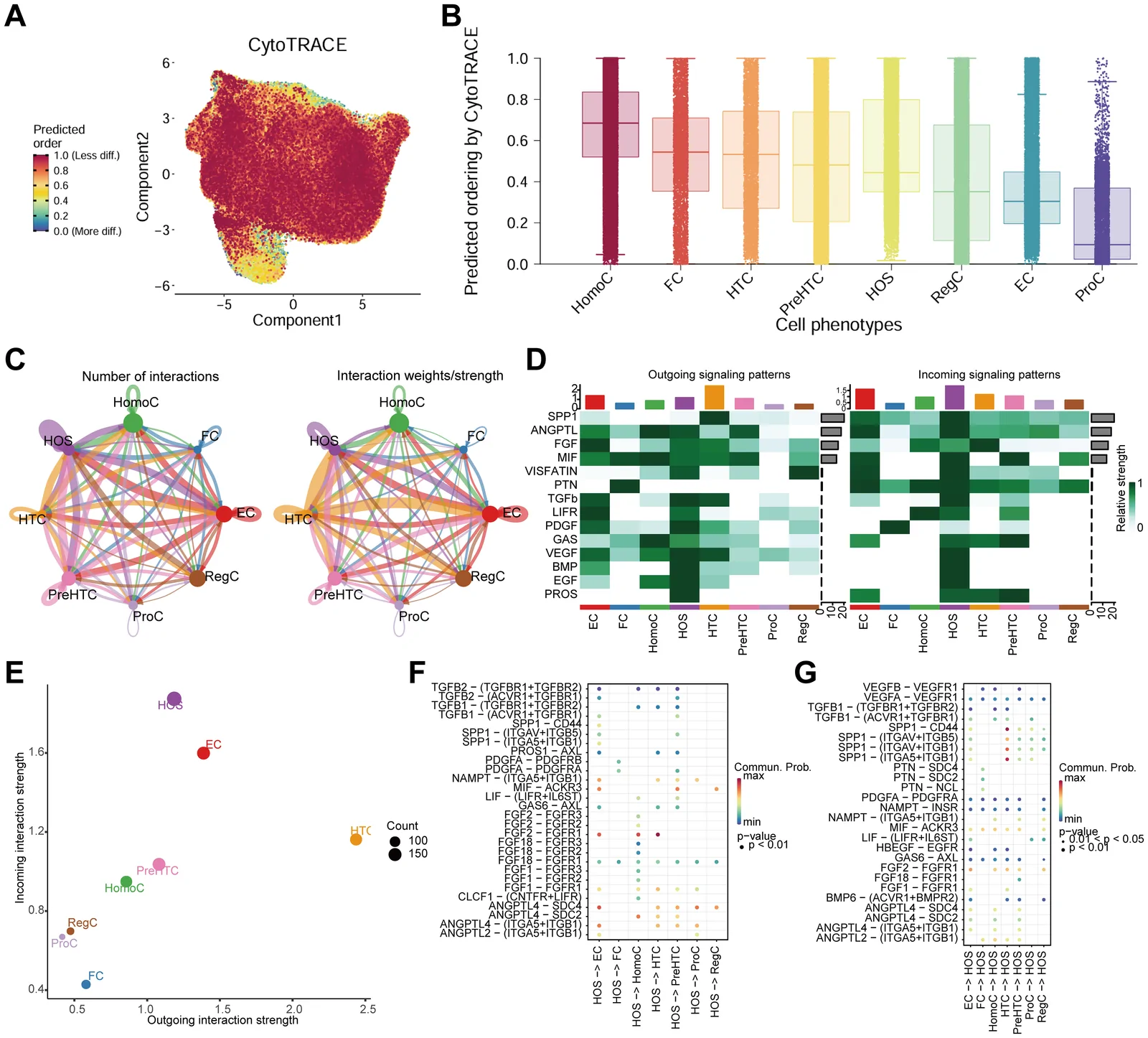
Cell–cell communication and CytoTRACE features of HOS/EC-enriched populations in OA cartilage. **(A)** CytoTRACE-inferred differentiation state of HOS cells. **(B)** Predicted ordering by CytoTRACE across cell phenotypes. **(C)** Network showing the number and strength of inferred interactions among cell populations. **(D)** Outgoing and incoming signaling patterns. **(E)** Scatter plot showing outgoing and incoming interaction strength across cell populations. **(F, G)** Bubble plots showing representative ligand–receptor interactions involving HOS-related communication. HOS was treated as a score-defined high-oxidative-stress cell group, whereas EC represents the annotated effector chondrocyte subpopulation.

Because the HOS density distribution overlapped with the EC-annotated region, we next examined the cell-state and communication features of the overall HOS population in the context of EC-associated oxidative stress. Here, HOS was treated as a score-defined high-oxidative-stress cell group, whereas EC represents the annotated effector chondrocyte subpopulation. HOS cells showed intermediate-to-high CytoTRACE scores, exceeding those of ECs, RegCs, and ProCs but not all annotated populations, suggesting a less differentiated or more transcriptionally active state (**Figures 4A, B**). CellChat analysis showed broad communication between HOS/EC-enriched cells and multiple chondrocyte populations by both interaction number and interaction strength (**Figure 4C**).

Signaling-pattern analysis suggested that HOS/EC-enriched cells functioned as both senders and receivers within the inferred communication network (**Figures 4D, E**). At the pathway level, HOS-related communication involved programs associated with inflammation, extracellular matrix remodeling, stress responses, and OA-relevant signaling, including SPP1, ANGPTL, FGF, MIF, TGF-β, VEGF, BMP, EGF, and PROS signaling (**Figures 4F, G**). These results suggest that HOS/EC-enriched cells may participate in multiple communication programs relevant to OA-associated inflammatory activation, matrix turnover, and oxidative stress-related tissue responses. These CellChat results were interpreted as inferred communication patterns rather than direct evidence of ligand–receptor activity.

### 3.5 Integrated hdWGCNA, machine learning, PPI, and SMR analyses prioritize EIF6 and YWHAB as candidate molecules associated with EC-related oxidative stress

**Figure 5:**
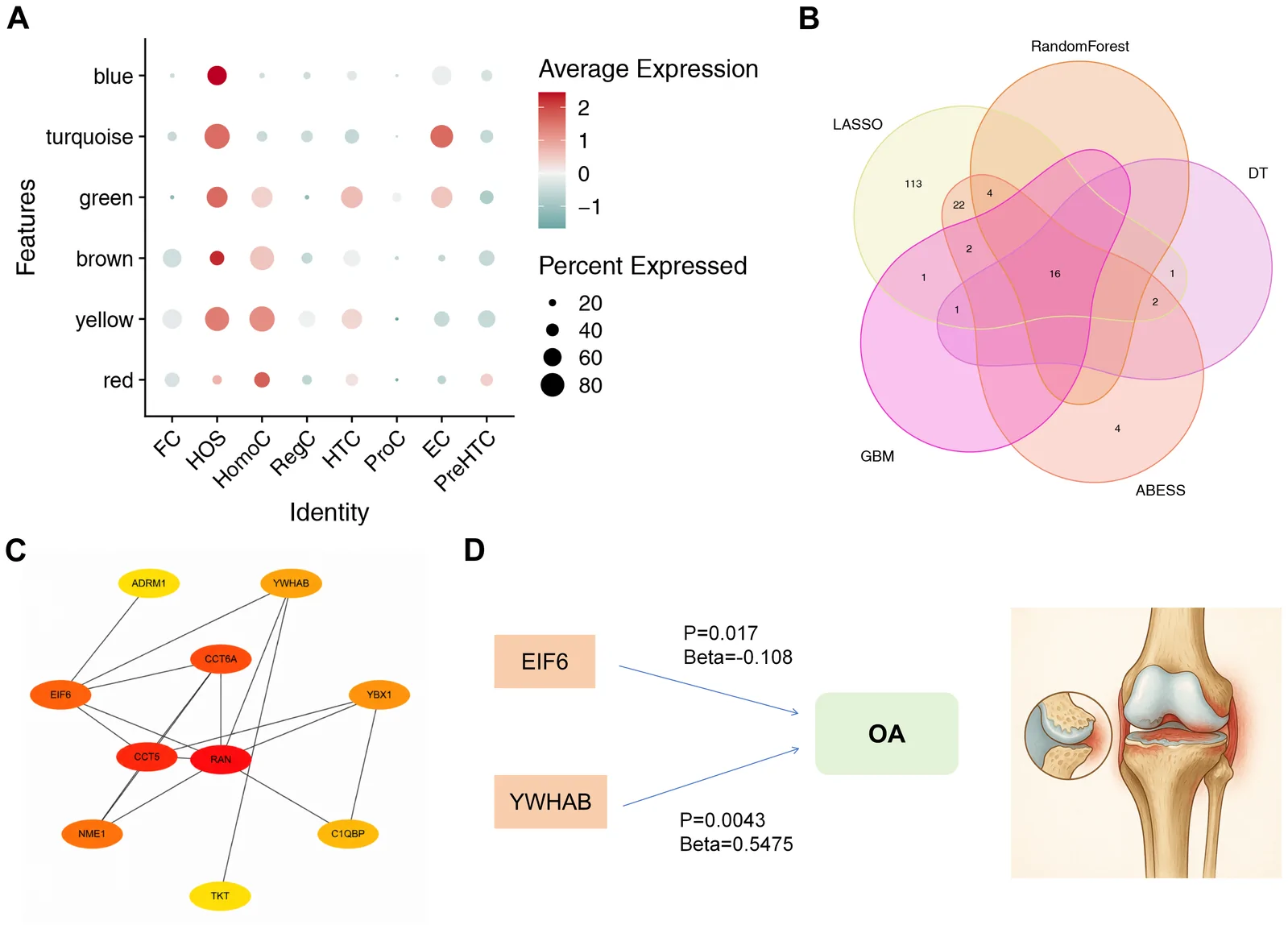
Prioritization of EIF6 and YWHAB as candidate molecules associated with EC-related oxidative stress in OA. **(A)** hdWGCNA module–cell type association analysis. **(B)** Venn diagram integrating five machine learning algorithms and identifying 16 overlapping genes. **(C)** PPI network of candidate genes, highlighting hub genes including EIF6 and YWHAB. **(D)** SMR analysis prioritizing EIF6 and YWHAB as supportive candidates. Because GTEx whole-blood eQTL data were used, SMR findings were interpreted as supportive genetic prioritization evidence rather than confirmatory causal evidence in joint tissues or ECs.

hdWGCNA was used to link co-expression structure with cell-type features and oxidative stress activity. Module–cell type association analysis identified modules related to specific annotated cell populations, including an EC-associated module (**Figure 5A**). Module construction, network visualization, and module–oxidative stress correlation results are shown in **Supplementary Figures 2A–C**. Enrichment analyses implicated protein homeostasis, RNA and protein localization, proteasome-related processes and energy metabolism (**Supplementary Figures 2D–F**).

We then applied five machine learning approaches, including random forest, LASSO, decision tree, GBM, and ABESS, to prioritize candidate genes. Sixteen genes were retained at the intersection of these algorithms (**Figure 5B**). Model performance analyses, including random forest error curves, feature-importance ranking, LASSO selection, AUC comparison, decision curve analysis, confusion matrix, and ROC analysis, are provided in **Supplementary Figures 3A–G**. These analyses were used for candidate prioritization rather than clinical model development.

The PPI network contained several highly connected genes, including RAN, CCT5, and CCT6A, while EIF6 and YWHAB were retained as candidates supported by the integrated prioritization strategy (**Figure 5C**). SMR analysis integrating OA GWAS summary statistics with GTEx whole-blood eQTL data prioritized EIF6 and YWHAB as supportive candidates (**Figure 5D**). Because the eQTL source was whole blood, these results were treated as supportive genetic prioritization evidence rather than proof of causality in joint tissues or ECs. Molecular docking suggested a possible EIF6–YWHAB interaction but was considered structural prediction only (**Supplementary Figure 2G**). Together, these analyses prioritized EIF6 and YWHAB as candidate molecules associated with EC-related oxidative stress.

### 3.6 EIF6 is associated with p47phox–NOX2-related ROS responses in an inflammatory effector chondrocyte-like model

Before functional validation, the identity of the inflammatory effector chondrocyte-like model was examined. qPCR analysis showed expression of chondrocyte-associated markers, including SOX9, ACAN, COL2A1, and COMP, and low baseline expression of vascular-lineage markers, including PECAM1/CD31, CDH5, VWF, and EMCN (**Supplementary Figures 5A, B**). IL-1β stimulation increased inflammatory and matrix-remodeling markers, including IL6, CXCL8, PTGS2, and MMP13 (**Supplementary Figure 5C**). Representative EC-associated markers, including KCNMA1, FST, and FGF1, also showed expression changes consistent with inflammatory EC-like activation (**Supplementary Figure 5D**). These results supported the use of C28/I2-derived cells as an inflammatory EC-like model for experimental validation.

The efficiency of EIF6 knockdown, YWHAB knockdown, EIF6 overexpression, and p47phox/NCF1 knockdown was confirmed before downstream analyses. qPCR and western blotting showed that siEIF6 reduced EIF6 expression, whereas siYWHAB reduced YWHAB expression (**Supplementary Figures 6A, B**). EIF6 overexpression increased EIF6 expression at both mRNA and protein levels (**Supplementary Figures 6C, D**). In the EIF6-overexpression setting, si-p47phox reduced p47phox/NCF1 expression while maintaining EIF6 overexpression (**Supplementary Figures 6E, F**).

**Figure 6:**
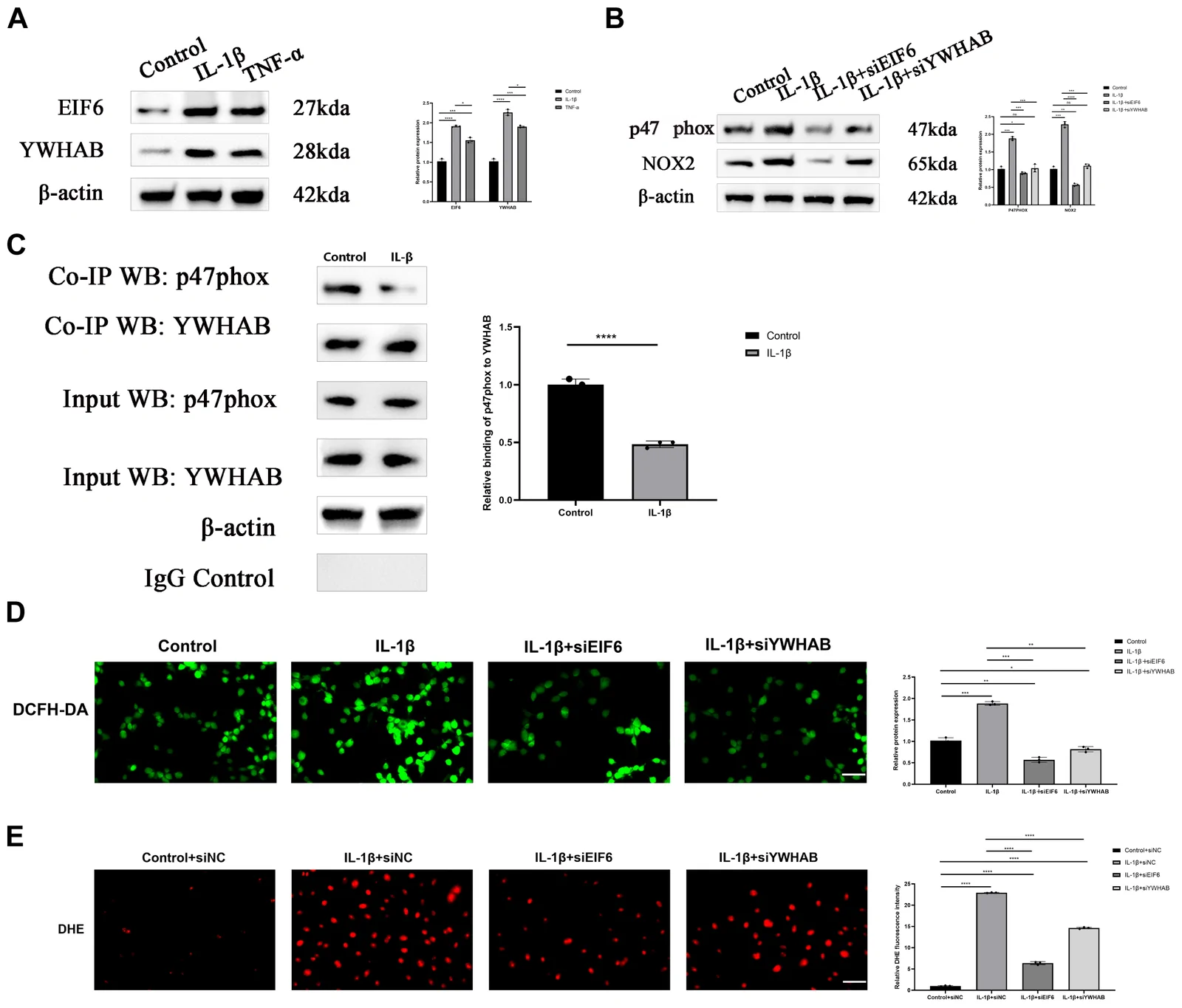
Experimental validation of EIF6-associated p47phox–NOX2 signaling in inflammatory effector chondrocyte-like cells. **(A)** Western blot analysis of EIF6 and YWHAB after IL-1β or TNF-α stimulation. **(B)** Effects of EIF6 or YWHAB knockdown on p47phox and NOX2 under IL-1β stimulation. **(C)** Co-immunoprecipitation of YWHAB and p47phox. Co-IP results were labeled as IP: YWHAB; WB: p47phox and IP: YWHAB; WB: YWHAB. **(D)** DCFH-DA staining showing intracellular ROS-related fluorescence; fluorescence was quantified as integrated density per field. **(E)** DHE staining as an additional ROS-related fluorescence assay; fluorescence was quantified as integrated density per field. Data are mean ± SEM; n = 3. *P < 0.05, **P < 0.01, ***P < 0.001, ****P < 0.0001.

To test the candidate molecules under inflammatory conditions, inflammatory EC-like cells were treated with IL-1β or TNF-α. Both stimuli increased EIF6 and YWHAB protein expression relative to control conditions (**Figure 6A**). Under IL-1β stimulation, p47phox and NOX2 were increased. Both EIF6 knockdown and YWHAB knockdown attenuated this induction, with EIF6 knockdown showing the stronger effect (**Figure 6B**). Basal-condition knockdown results are shown in **Supplementary Figure 4A**. These data indicate that both EIF6 and YWHAB were associated with IL-1β-induced p47phox–NOX2 expression, with EIF6 showing a more pronounced association in this setting.

Co-immunoprecipitation using an anti-YWHAB antibody showed that less p47phox was co-precipitated with YWHAB after IL-1β stimulation, while input protein levels remained broadly comparable (**Figure 6C**). This suggests that inflammatory stimulation may alter relative YWHAB–p47phox co-precipitation, although further quantitative interaction assays are required to confirm changes in binding dynamics.

DCFH-DA staining showed that IL-1β increased intracellular ROS-related fluorescence. Both EIF6 knockdown and YWHAB knockdown reduced IL-1β-induced ROS-related fluorescence, with EIF6 knockdown showing the stronger reduction (**Figure 6D**). Consistently, DHE staining showed increased ROS-related fluorescence after IL-1β stimulation, whereas EIF6 and YWHAB knockdown attenuated this response, again with a stronger effect observed after EIF6 knockdown (**Figure 6E**). Additional p65 immunofluorescence showed increased p65 signal after IL-1β stimulation and EIF6 overexpression, supporting an association with NF-κB-related inflammatory activation (**Supplementary Figure 4B**). Overall, EIF6 and YWHAB were both associated with p47phox–NOX2 expression and ROS-related fluorescence in inflammatory EC-like cells, whereas the Co-IP results suggested that YWHAB may also be involved in p47phox interaction dynamics.

### 3.7 EIF6 manipulation is associated with altered p47phox and TGF-β responses in DMM-induced OA tissues

We next examined whether the *in vitro* findings were reflected in OA-like joint tissues. The efficiency of *in vivo* EIF6 knockdown, EIF6 overexpression, and p47phox knockdown was confirmed by IHC analysis in the corresponding DMM groups. EIF6 IHC staining confirmed reduced EIF6 expression in the DMM + siEIF6 group compared with the DMM + siNC group (**Supplementary Figure 7A**). EIF6 overexpression increased EIF6 staining in DMM tissues (**Supplementary Figure 7B**), and p47phox knockdown reduced p47phox staining in the DMM + EIF6-OE + si-p47phox group (**Supplementary Figure 7C**).

**Figure 7:**
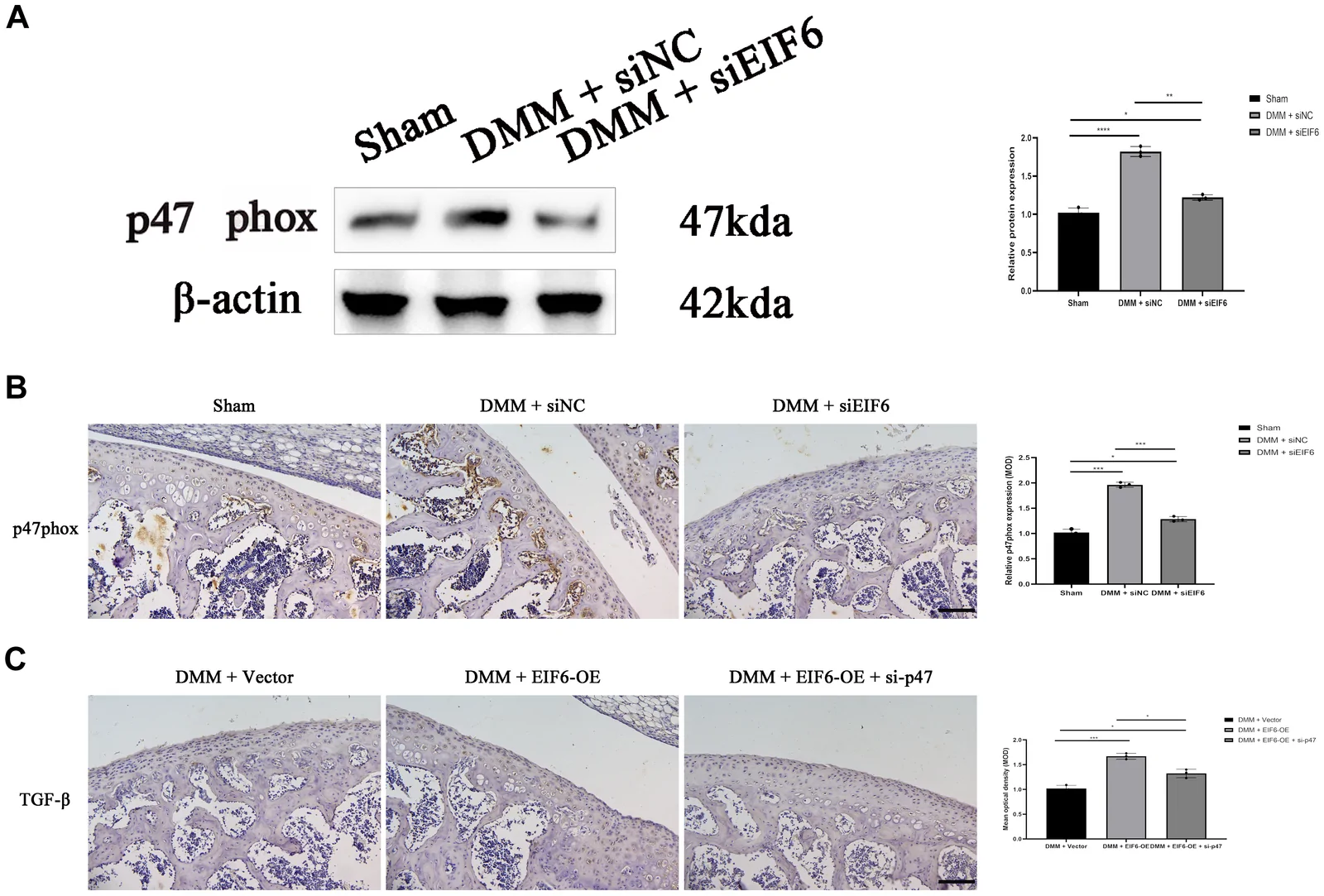
Tissue-level validation of p47phox and TGF-β responses in DMM-induced OA mice. **(A)** Western blot analysis of p47phox in Sham, DMM + siNC, and DMM + siEIF6 groups. **(B)** Immunohistochemical staining and quantification of p47phox. **(C)** Immunohistochemical staining and quantification of TGF-β in DMM + Vector, DMM + EIF6-OE, and DMM + EIF6-OE + si-p47phox groups. Data are mean ± SEM; n = 3. *P < 0.05, **P < 0.01, ***P < 0.001, ****P < 0.0001.

In the DMM model, p47phox expression increased relative to Sham controls, while EIF6 knockdown reduced DMM-induced p47phox upregulation (**Figure 7A**). This direction was consistent with the *in vitro* EC-like findings. Immunohistochemistry confirmed stronger p47phox staining in the DMM + siNC group and reduced staining in the DMM + siEIF6 group, although staining remained above the Sham level (**Figure 7B**). EIF6 overexpression increased TGF-β staining in DMM tissues, and p47phox knockdown partly reduced this response without fully restoring it to baseline (**Figure 7C**). These *in vivo* data provide tissue-level support for an association between EIF6 manipulation and p47phox/TGF-β-related responses, but they do not establish EIF6 as an independent driver of OA progression.

## 4 Discussion

This study used a single-cell framework to examine oxidative stress activity in OA cartilage datasets and to prioritize candidate molecules linked to effector chondrocyte (EC)-associated stress features. The major findings are that the annotated EC population was increased in OA samples, showed relatively high oxidative stress activity, and formed the largest component of high oxidative stress (HOS) cells. HOS/EC-enriched cells were predicted to participate in inflammatory, growth-factor-related, stress-associated, and matrix-remodeling communication programs. Integrated computational prioritization highlighted EIF6 and YWHAB as candidate molecules associated with EC-related oxidative stress. *In vitro* validation linked EIF6/YWHAB-associated changes to p47phox–NOX2 expression and ROS-related fluorescence in an inflammatory EC-like model, whereas DMM-induced OA tissues provided tissue-level support for EIF6-associated p47phox/TGF-β responses.

The enrichment of the annotated EC population in OA cartilage and in the HOS compartment is an important observation. OA is increasingly regarded as a whole-joint disease, with pathological contributions from cartilage, synovium, subchondral bone, meniscus, and periarticular tissues (Loeser et al., 2012). Inflammatory changes in OA synovium may contribute to symptoms and structural disease features (Scanzello and Goldring, 2012). Our single-cell analysis extends this view by showing that oxidative stress activity was not uniformly distributed across cartilage cell states. Instead, the EC population showed relatively high oxidative stress scores, was increased in OA samples, and accounted for the largest proportion of HOS cells. In addition, the proportion of ECs among HOS cells increased from normal to OA cartilage, and HOS density was concentrated near the EC-enriched region in the UMAP space. These results suggest that EC-associated transcriptional states are closely linked to oxidative stress activity in OA cartilage datasets.

The EC-associated oxidative stress signal described here should be interpreted as a chondrocyte-state-associated feature defined by single-cell chondrocyte subpopulation annotation. Consistently, the inflammatory EC-like model expressed chondrocyte-associated markers and displayed IL-1β-induced inflammatory and matrix-remodeling activation, supporting its use for downstream validation.

The predicted communication profile of HOS/EC-enriched cells provides additional biological context. CellChat analysis linked HOS/EC-enriched cells to signaling programs involving SPP1, ANGPTL, FGF, MIF, TGF-β, VEGF, BMP, EGF, PROS, and other OA-relevant pathways. These pathways are consistent with recognized OA processes, including inflammatory activation, growth-factor signaling, extracellular matrix remodeling, and osteochondral tissue responses (Loeser et al., 2012; Scanzello and Goldring, 2012). EC-associated oxidative stress may therefore represent a chondrocyte activation state connected with inflammatory and matrix-remodeling processes (Ji et al., 2019; Swahn et al., 2023). However, ligand–receptor analysis from scRNA-seq is inferential. These communication patterns should be interpreted as hypothesis-generating and require validation in anatomically defined joint tissues or spatially resolved datasets.

Integrated prioritization highlighted EIF6 and YWHAB as candidate molecules associated with EC-related oxidative stress. The computational strategy combined hdWGCNA, machine learning, PPI network analysis, SMR, and molecular docking. This multilayered approach reduced dependence on any single prioritization method and allowed the identification of candidates supported by multiple lines of evidence. Nevertheless, these analyses should be interpreted as prioritization rather than definitive causal inference. This is especially relevant for SMR, because GTEx whole-blood eQTL data were used and do not directly represent joint tissues or ECs. Thus, the SMR results provide supportive genetic evidence for candidate selection rather than confirmatory causal evidence in OA cartilage.

EIF6 and YWHAB showed distinct but related experimental patterns in the inflammatory EC-like model. IL-1β and TNF-α increased EIF6 and YWHAB expression, suggesting that both molecules are responsive to inflammatory stimulation. Under IL-1β stimulation, p47phox and NOX2 were increased, whereas EIF6 knockdown and YWHAB knockdown attenuated this induction. The effect of EIF6 knockdown was stronger than that of YWHAB knockdown. DCFH-DA and DHE staining further showed that EIF6 or YWHAB knockdown reduced ROS-related fluorescence, again with a stronger effect after EIF6 knockdown. These data support a closer association between EIF6 and p47phox–NOX2-related ROS responses under inflammatory EC-like conditions.

The role of YWHAB appears more complex. The 14-3–3 family has biological relevance in OA-related signaling; for example, 14-3-3ε has been reported as an intracellular component of the TNFR2 complex with protective effects in OA models (Fu et al., 2021b). In our study, YWHAB knockdown attenuated IL-1β-induced p47phox–NOX2 expression and ROS-related fluorescence, but the magnitude of this effect was weaker than that of EIF6 knockdown. Co-immunoprecipitation further suggested reduced YWHAB–p47phox association after IL-1β stimulation. These findings do not support a simple linear EIF6–YWHAB–p47phox pathway. Rather, they suggest that EIF6 is more closely associated with p47phox–NOX2 expression, whereas YWHAB may be involved in p47phox interaction dynamics or inflammatory remodeling of regulatory protein complexes.

The p47phox–NOX2 axis provides a biologically plausible link between EIF6-associated changes and ROS-related responses. p47phox is a key organizer of the phagocyte NOX2 NADPH oxidase complex and is required for activation-associated oxidase assembly (El-Benna et al., 2009). NADPH oxidases have been implicated in OA-related oxidative stress, and their inhibition can attenuate OA development in experimental models (Han et al., 2022). Recent OA studies have further highlighted oxidative stress as a therapeutically relevant process in OA, including NOX2/ROS-related chondrocyte injury (Ni et al., 2021), mitochondrial-targeted intervention (Zhu et al., 2026), modulation of mitochondrial homeostasis within oxi-inflammatory aging networks (Zhang et al., 2026), and Nrf2/NF-κB regulation (Zhang et al., 2025). Consistent with this biology, IL-1β increased p47phox and NOX2 expression in inflammatory EC-like cells, while EIF6 knockdown attenuated these proteins and reduced ROS-related fluorescence. p47phox-related ROS production has also been linked to oxidative stress-induced chondrocyte damage (Shin et al., 2020).

The additional validation experiments strengthened the interpretation of the *in vitro* findings. qPCR validation supported the chondrocyte-like identity and inflammatory activation of the EC-like model. Knockdown and overexpression controls confirmed that siEIF6, siYWHAB, EIF6 overexpression, and si-p47phox/NCF1 achieved the intended gene manipulation before downstream functional assays. These controls are important because they reduce the possibility that the observed changes in p47phox, NOX2, and ROS-related fluorescence were due to ineffective or nonspecific manipulation. However, DCFH-DA and DHE are fluorescence-based ROS-related probes and do not directly identify a single ROS species or prove NOX2 enzymatic activation. Future studies should further examine p47phox phosphorylation, cytoplasmic-to-membrane translocation, NOX2 complex assembly, and direct NADPH oxidase activity in primary joint effector chondrocytes.

The DMM model provided tissue-level support for the *in vitro* observations. DMM is a well-established model of surgically induced OA that causes joint instability and progressive degeneration (Glasson et al., 2007). In this model, p47phox expression was increased in OA-like joints and was reduced by EIF6 knockdown. IHC validation confirmed the efficiency of *in vivo* EIF6 knockdown, EIF6 overexpression, and p47phox knockdown in the corresponding DMM groups. EIF6 overexpression also increased TGF-β staining, whereas p47phox knockdown partially reduced this response. These findings support an association between EIF6 manipulation and p47phox/TGF-β-related tissue changes. However, they should not be interpreted as evidence that EIF6 alone drives OA progression. The *in vivo* experiments were designed to provide tissue-level validation of EIF6-associated p47phox/TGF-β responses rather than to establish EC-specific genetic causality.

This study has several strengths. It combines single-cell oxidative stress scoring with cell communication analysis, network-based candidate prioritization, and validation in both an inflammatory EC-like model and OA-like tissues. Multiple scoring algorithms were used to reduce dependence on any single oxidative stress metric. The HOS definition provided a practical way to focus on cells with the most prominent oxidative stress activity, and normal-versus-OA HOS composition analysis further supported the OA enrichment of EC-associated oxidative stress features. The integration of hdWGCNA, machine learning, PPI, SMR, and docking allowed EIF6 and YWHAB to be prioritized from complementary computational perspectives. Experimental validation further connected these candidates to p47phox–NOX2 expression, ROS-related fluorescence, and tissue-level p47phox/TGF-β responses.

The study also has important limitations. First, EC identity was inferred transcriptomically from public scRNA-seq datasets, and the EC-like *in vitro* model may not fully recapitulate primary effector chondrocytes in native OA cartilage. Second, SMR relied on GTEx whole-blood eQTL data rather than joint- or EC-specific eQTL resources, and therefore should be interpreted as supportive prioritization evidence rather than confirmatory causal evidence. Third, ligand–receptor communication analysis was computationally inferred and requires spatial or functional validation. Finally, the p47phox–NOX2 axis was assessed mainly through protein expression, co-immunoprecipitation, and ROS-related fluorescence assays. Spatial transcriptomics, primary joint effector chondrocytes, effector chondrocyte-specific genetic manipulation, and direct assessment of NOX2 activation will be needed to refine and validate the proposed pathway.

## 5 Conclusion

In summary, this study identifies an annotated EC-enriched oxidative stress state in OA cartilage datasets and prioritizes EIF6 and YWHAB as candidate molecules associated with EC-related oxidative stress. Experimental data support an association between EIF6 and p47phox–NOX2-related ROS responses under inflammatory EC-like conditions, with consistent tissue-level evidence from DMM-induced OA joints. These findings provide a cell-state-resolved framework for understanding EC-associated oxidative stress in OA and support further mechanistic investigation of EIF6-related p47phox–NOX2 signaling.

## Data Availability

Public single-cell RNA-seq datasets analyzed in this study are available from the Gene Expression Omnibus under accession numbers GSE220243 and GSE169454. Osteoarthritis GWAS summary statistics are available from the GWAS Catalog under accession number GCST005812. GTEx v8 whole-blood eQTL data were used for SMR analysis. The remaining data generated or analyzed during this study are available from the corresponding author upon reasonable request.

## References

[B19] Aibar S. González-Blas C. B. Moerman T. Huynh-Thu V. A. Imrichova H. Hulselmans G. . (2017). SCENIC: single-cell regulatory network inference and clustering. Nat. Methods 14, 1083–1086. doi: 10.1038/nmeth.4463 28991892 PMC5937676

[B20] Andreatta M. Carmona S. J. (2021). UCell: robust and scalable single-cell gene signature scoring. Comput. Struct. Biotechnol. J. 19, 3796–3798. doi: 10.1016/j.csbj.2021.06.043 34285779 PMC8271111

[B22] Barbie D. A. Tamayo P. Boehm J. S. Kim S. Y. Moody S. E. Dunn I. F. . (2009). Systematic RNA interference reveals that oncogenic KRAS-driven cancers require TBK1. Nature 462, 108–112. doi: 10.1038/nature08460 19847166 PMC2783335

[B27] Breiman L. (2001). Random forests. Mach. Learn. 45, 5–32. doi: 10.1023/a:1010933404324 41886696

[B28] Breiman L. Friedman J. H. Olshen R. A. Stone C. J. (1984). Classification and regression trees (CART). Biometrics 40, 358. doi: 10.1201/9781315139470

[B10] Buniello A. MacArthur J. A. L. Cerezo M. Harris L. W. Hayhurst J. Malangone C. . (2019). The NHGRI-EBI GWAS Catalog of published genome-wide association studies, targeted arrays and summary statistics 2019. Nucleic Acids Res. 47, D1005–D1012. doi: 10.1093/nar/gky1120 30445434 PMC6323933

[B12] Consortium GT (2020). The GTEx Consortium atlas of genetic regulatory effects across human tissues. Science 369, 1318–1330. doi: 10.1101/787903 32913098 PMC7737656

[B43] El-Benna J. Dang P.-C. Gougerot-Pocidalo M.-A. Marie J.-C. Braut-Boucher F. (2009). p47phox, the phagocyte NADPH oxidase/NOX2 organizer: structure, phosphorylation and implication in diseases. Exp. Mol. Med. 41, 217–225. doi: 10.3858/emm.2009.41.4.058 19372727 PMC2679237

[B21] Foroutan M. Bhuva D. D. Lyu R. Horan K. Cursons J. Davis M. J. (2018). Single sample scoring of molecular phenotypes. BMC Bioinf. 19, 404. doi: 10.1186/s12859-018-2435-4 30400809 PMC6219008

[B31] Friedman J. H. (2001). Greedy function approximation: a gradient boosting machine. Ann. Stat. 29, 1189–1232. doi: 10.1214/aos/1013203451

[B8] Fu W. Hettinghouse A. Chen Y. Hu W. Ding X. Chen M. . (2021a). 14-3-3ε is an intracellular component of TNFR2 receptor complex and its activation protects against osteoarthritis. Ann. Rheum Dis. 80, 1615–1627. doi: 10.1136/annrheumdis-2021-220000 34226187 PMC8595573

[B42] Fu W. Hettinghouse A. Chen Y. Hu W. Ding X. Chen M. . (2021b). 14-3-3ε is an intracellular component of TNFbib2 receptor complex and its activation protects against osteoarthritis. Ann. Rheum Dis. 80, 1615–1627. doi: 10.1136/annrheumdis-2021-220000 34226187 PMC8595573

[B36] Gan K. Wu W. Li J. Xu D. Liu Y. Bi M. . (2022). Positive feedback loop of lncRNA FAM201A/miR-146a-5p/POU2F1 regulates IL-1β-induced chondrocyte injury *in vitro*. Mol. Med. Rep. 25, 20. doi: 10.3892/mmr.2021.12536 34796909 PMC8628288

[B41] Glasson S. S. Blanchet T. J. Morris E. A. (2007). The surgical destabilization of the medial meniscus (DMM) model of osteoarthritis in the 129/ SvEv mouse. Osteoarthritis Cartilage 15, 1061–1069. doi: 10.1016/j.joca.2007.03.006 17470400

[B24] Gulati G. S. Sikandar S. S. Wesche D. J. Manjunath A. Bharadwaj A. Berger M. J. . (2020). Single-cell transcriptional diversity is a hallmark of developmental potential. Science 367, 405–411. doi: 10.1126/science.aax0249 31974247 PMC7694873

[B4] Han J. Park D. Park J. Han S. (2022). Inhibition of NADPH oxidases prevents the development of osteoarthritis. Antioxidants 11, 16. doi: 10.3390/antiox11122346 36552552 PMC9774355

[B23] Hänzelmann S. Castelo R. Guinney J. (2013). GSVA: gene set variation analysis for microarray and RNA-seq data. BMC Bioinf. 14, 7. doi: 10.1186/1471-2105-14-7 PMC3618321 23323831

[B14] Hao Y. Stuart T. Kowalski M. H. Choudhary S. Hoffman P. Hartman A. . (2024). Dictionary learning for integrative, multimodal and scalable single-cell analysis. Nat. Biotechnol. 42, 293–304. doi: 10.1038/s41587-023-01767-y 37231261 PMC10928517

[B1] Hunter D. Bierma-Zeinstra S. (2019). Osteoarthritis. Lancet 393, 1745–1759. doi: 10.1016/j.berh.2011.11.008 31034380

[B5] Ji Q. Zheng Y. Zhang G. Hu Y. Fan X. Hou Y. . (2019). Single-cell RNA-seq analysis reveals the progression of human osteoarthritis. Ann. Rheum Dis. 78, 100–110. doi: 10.1136/annrheumdis-2017-212863 30026257 PMC6317448

[B25] Jin S. Guerrero-Juarez C. F. Zhang L. Chang I. Ramos R. Kuan C.-H. . (2021). Inference and analysis of cell-cell communication using CellChat. Nat. Commun. 12, 1088. doi: 10.1038/s41467-021-21246-9 33597522 PMC7889871

[B37] Kalyanaraman B. Darley-Usmar V. Davies K. J. A. Dennery P. A. Forman H. J. Grisham M. B. . (2012). Measuring reactive oxygen and nitrogen species with fluorescent probes: challenges and limitations. Free Radical Biol. Med. 52, 1–6. doi: 10.1016/j.freeradbiomed.2011.09.030 22027063 PMC3911769

[B16] Korsunsky I. Millard N. Fan J. Slowikowski K. Zhang F. Wei K. . (2019). Fast, sensitive and accurate integration of single-cell data with Harmony. Nat. Methods 16, 1289–1296. doi: 10.1038/s41592-019-0619-0 31740819 PMC6884693

[B39] Laemmli U. K. (1970). Cleavage of structural proteins during the assembly of the head of bacteriophage T4. Nature. 227 (5259), 680–685. doi: 10.1038/227680a0 5432063

[B26] Langfelder P. Horvath S. (2008). WGCNA: an R package for weighted correlation network analysis. BMC Bioinf. 9, 559. doi: 10.1186/1471-2105-9-559 19114008 PMC2631488

[B18] Liberzon A. Birger C. Thorvaldsdóttir H. Ghandi M. Mesirov J. P. Tamayo P. (2015). The molecular signatures database hallmark gene set collection. Cell Syst. 1, 417–425. doi: 10.1016/j.cels.2015.12.004 26771021 PMC4707969

[B2] Loeser R. Goldring S. Scanzello C. Goldring M. (2012). Osteoarthritis: a disease of the joint as an organ. Arthritis Rheumatol. 64, 1697–1707. doi: 10.1002/art.34453 22392533 PMC3366018

[B15] Luecken M. D. Theis F. J. (2019). Current best practices in single‐cell RNA‐seq analysis: a tutorial. Mol. Syst. Biol. 15, e8746. doi: 10.15252/msb.20188746 31217225 PMC6582955

[B17] McInnes L. Healy J. Saul N. Großberger L. (2018). UMAP: uniform manifold approximation and projection. J. Open Source Software 3 (29), 861. doi: 10.21105/joss.00861

[B44] Ni S. Li D. Wei H. Miao K.-S. Zhuang C. (2021). PPARγ attenuates interleukin-1β-induced cell apoptosis by inhibiting NOX2/ROS/p38MAPK activation in osteoarthritis chondrocytes. Oxid. Med. Cell. Longevity 2021, 5551338. doi: 10.1155/2021/5551338 PMC8112933 34055194

[B7] Scanzello C. R. Goldring S. R. (2012). The role of synovitis in osteoarthritis pathogenesis. Bone 51, 249–257. doi: 10.1016/j.bone.2012.02.012 22387238 PMC3372675

[B38] Schneider C. A. Rasband W. S. Eliceiri K. W. (2012). NIH Image to ImageJ: 25 years of image analysis. Nat. Methods 9, 671–675. doi: 10.1038/nmeth.2089 22930834 PMC5554542

[B33] Shannon P. (2003). Cytoscape: a software environment for integrated models of biomolecular interaction networks. Genome Res. 13, 2498–2504. doi: 10.1101/gr.1239303 14597658 PMC403769

[B48] Shin H. J. Park H. Shin N. Kwon H. H. Yin Y. Hwang J.-A. . (2020). p47phox siRNA-loaded PLGA nanoparticles suppress ROS/oxidative stress-induced chondrocyte damage in osteoarthritis. Polymers 12, 443. doi: 10.3390/polym12020443 32069893 PMC7077645

[B13] Stuart T. Butler A. Hoffman P. Hafemeister C. Papalexi E. Mauck W. M. . (2019). Comprehensive integration of single-cell data. Cell. 177, 1888–1902.e21. doi: 10.1016/j.cell.2019.05.031 31178118 PMC6687398

[B9] Swahn H. Li K. Duffy T. Olmer M. D'Lima D. D. Mondala T. S. . (2022). Senescent cell population with ZEB1 transcription factor as its main regulator promotes osteoarthritis in cartilage and meniscus. Ann. Rheum Dis. 82, 403–415. doi: 10.1136/ard-2022-223227 36564153 PMC10076001

[B6] Swahn H. Li K. Duffy T. Olmer M. D'Lima D. Mondala T. . (2023). Senescent cell population with ZEB1 transcription factor as its main regulator promotes osteoarthritis in cartilage and meniscus. Ann. Rheum Dis. 82, 403–415. doi: 10.1136/ard-2022-223227 36564153 PMC10076001

[B32] Szklarczyk D. Nastou K. Koutrouli M. Kirsch R. Mehryary F. Hachilif R. . (2025). The STRING database in 2025: protein networks with directionality of regulation. Nucleic Acids Res. 53, D730–D737. doi: 10.1093/nar/gkae1113 39558183 PMC11701646

[B29] Tibshirani R. (1996). Regression shrinkage and selection via the lasso. J. R. Stat. Society: Ser. B. (Methodological) 58, 267–289. doi: 10.1111/j.2517-6161.1996.tb02080.x 40046247

[B40] Towbin H. Staehelin T. Gordon J. (1979). Electrophoretic transfer of proteins from polyacrylamide gels to nitrocellulose sheets: procedure and some applications. Proc. Natl. Acad. Sci. 76, 4350–4354. doi: 10.1073/pnas.76.9.4350 388439 PMC411572

[B35] Trott O. Olson A. J. (2009). AutoDock Vina: improving the speed and accuracy of docking with a new scoring function, efficient optimization, and multithreading. J. Comput. Chem. 31, 21334. doi: 10.1002/jcc.21334 19499576 PMC3041641

[B3] Zahan O.-M. Serban O. Gherman C. Fodor D. (2020). The evaluation of oxidative stress in osteoarthritis. Med. Pharm. Rep. 93, 12–22. doi: 10.15386/mpr-1422 32133442 PMC7051818

[B11] Zengini E. Hatzikotoulas K. Tachmazidou I. Steinberg J. Hartwig F. P. Southam L. . (2018). Genome-wide analyses using UK Biobank data provide insights into the genetic architecture of osteoarthritis. Nat. Genet. 50, 549–558. doi: 10.1038/s41588-018-0079-y 29559693 PMC5896734

[B46] Zhang S. O. Gu Z. Q. Ruan J. Zhang J. Chen H. (2026). Mitochondrial-targeted therapy for osteoarthritis: challenges and opportunities from basic research to clinical translation. Front. Immunol. 17, 1696120. doi: 10.3389/fimmu.2026.1696120 41798918 PMC12963063

[B47] Zhang Z. Wang X. Fan P. Cheng D. Dang J. Dan X. . (2025). Coupling mitochondrial homeostasis to oxi-inflamm-aging network disruption via peptide-functionalized nanocomposite hydrogel for osteoarthritis intervention. Adv. Mater. 38, e11804. doi: 10.1002/adma.202511804 41450276

[B45] Zhu L. Shen H. Huang C. Jiang H. Yan Z. Pan X. . (2026). Asperuloside-mediated activation of Nrf2 inhibits the NF-κB pathway and suppresses osteoarthritis progression. Phytother. Res. 40 (5), 3027–3046. doi: 10.1002/ptr.70289 41823031

[B30] Zhu J. Wang X. Hu L. Huang J. Jiang K. Zhang Y. . (2022). abess: a fast best-subset selection library in Python and R. J. Mach. Learn. Res. 23, Article 202.

[B34] Zhu Z. Zhang F. Hu H. Bakshi A. Robinson M. R. Powell J. E. . (2016). Integration of summary data from GWAS and eQTL studies predicts complex trait gene targets. Nat. Genet. 48, 481–487. doi: 10.1038/ng.3538 27019110

