# Supplementary material for: Integrated single-cell and experimental validation links effector chondrocyte-associated oxidative stress to EIF6-related p47phox–NOX2 signaling in osteoarthritis
Source: Front Physiol. 2026 Aug 7;17:1882484. doi: 10.3389/fphys.2026.1882484 (PMC13494273; doi:10.3389/fphys.2026.1882484)
Supplement: Supplementary file 1 [file SupplementaryFile1.docx]

Supplementary Material

# 1 Supplementary Figures

**Supplementary Figure S1. Additional oxidative stress scoring analyses.**

(A) Dot plot showing oxidative stress activity across annotated cell populations using multiple single-cell gene-set scoring methods, including AUCell, UCell, singscore, ssGSEA, AddModuleScore, and the integrated scoring approach. (B) Ridge plots showing the distribution of integrated oxidative stress scores across annotated cell populations. (C) Density distribution of integrated oxidative stress scores among annotated cell populations. (D, E) UMAP visualization of integrated oxidative stress scores in normal and OA samples, respectively.

**Supplementary Figure S2. hdWGCNA and molecular docking analyses supporting candidate gene prioritization.**

(A) hdWGCNA dendrogram and module color assignment. (B) Network visualization of representative co-expression modules identified by hdWGCNA. (C) Ranking analysis of genes associated with oxidative stress-related module features. (D–F) Functional enrichment analyses of module-related genes, including Disease Ontology, KEGG pathway, and Gene Ontology enrichment analyses. (G) Molecular docking analysis supporting the predicted interaction between EIF6 and YWHAB.

**Supplementary Figure S3. Machine learning analyses for candidate molecule screening.**

(A) Random forest error curve. (B) Feature-importance ranking from the random forest model. (C) LASSO regression analysis for candidate gene selection. (D) Comparative AUC performance of different machine learning models. (E) Decision curve analysis. (F) Confusion matrix of model performance. (G) ROC curve analysis comparing different machine learning models.

**Supplementary Figure S4. Additional experimental validation of EIF6/YWHAB-related signaling.**
(A) Western blot analysis showing the effects of EIF6 or YWHAB knockdown under basal conditions. (B) Representative p65 immunofluorescence images showing inflammatory activation after IL-1β stimulation and EIF6 overexpression. Quantitative data are presented as mean ± SEM; n ≥ 3. *P < 0.05, **P < 0.01, ***P < 0.001.

Supplementary Figure S5. qPCR validation of the inflammatory effector chondrocyte-like model. (A) Chondrocyte-associated marker expression. (B) Low vascular-lineage marker expression. (C) IL-1β-induced inflammatory and matrix-remodeling marker expression. (D) Representative EC-associated marker expression after IL-1β stimulation.

Supplementary Figure S6. Validation of in vitro gene manipulation efficiency. (A,B) qPCR and western blot validation of EIF6 and YWHAB knockdown efficiency. (C,D) qPCR and western blot validation of EIF6 overexpression efficiency. (E,F) qPCR and western blot validation of p47phox/NCF1 knockdown in the EIF6-overexpression setting.

Supplementary Figure S7. Validation of in vivo gene manipulation efficiency in DMM-induced OA tissues. (A) EIF6 IHC validation of siEIF6-mediated knockdown. (B) EIF6 IHC validation of EIF6 overexpression. (C) p47phox IHC validation of si-p47phox-mediated knockdown.

# 2 **Supplementary Methods**

**2.1 Study overview**

This study integrated public single-cell transcriptomic datasets, oxidative stress scoring, cell–cell communication inference, high-dimensional weighted gene co-expression network analysis (hdWGCNA), machine learning, protein–protein interaction (PPI) network analysis, summary-data-based Mendelian randomization (SMR), molecular docking, and experimental validation to investigate effector chondrocyte (EC)-associated oxidative stress features in osteoarthritis (OA). ECs refer to effector chondrocytes throughout this study. Computational analyses were used to identify candidate molecules associated with EC-related oxidative stress, while cellular and animal experiments were performed to evaluate EIF6/YWHAB-associated p47phox–NOX2 signaling under inflammatory and OA-like conditions.

**2.2 Public datasets**

OA-related human cartilage single-cell RNA sequencing (scRNA-seq) datasets were downloaded from the Gene Expression Omnibus (GEO), including GSE220243 and GSE169454. The combined dataset included 18 human cartilage samples, consisting of nine normal samples and nine OA samples. OA genome-wide association study (GWAS) summary statistics were obtained from the GWAS Catalog dataset GCST005812. Whole-blood expression quantitative trait locus (eQTL) summary data were obtained from GTEx v8 and used for SMR analysis. Because all human transcriptomic and GWAS/eQTL data were obtained from public databases, no additional human participant recruitment was performed in this study.

**2.3 scRNA-seq quality control, normalization, integration, and clustering**

scRNA-seq analysis was performed in R using Seurat. Raw or processed gene expression matrices were imported into Seurat objects. Cells were filtered according to standard quality-control indicators, including the number of detected genes, total UMI/transcript counts, and mitochondrial gene expression percentage. Cells with low transcript complexity, unusually high transcript counts, or high mitochondrial gene proportions were excluded according to dataset-specific quality-control distributions.

After quality control, gene expression matrices were normalized. Highly variable genes were identified and used for dimensionality reduction. Principal component analysis was performed, and significant principal components were selected for downstream clustering. To reduce batch effects across datasets and samples, Harmony was applied for integration and correction. Neighbor graphs were generated using Seurat FindNeighbors, and clusters were identified using FindClusters. Uniform manifold approximation and projection (UMAP) was used for two-dimensional visualization of the integrated single-cell landscape.

**2.4 Cell-type annotation**

Cell clusters were annotated according to representative marker genes identified by Seurat FindAllMarkers and by comparison with previously reported cartilage cell markers. The main annotated populations included effector chondrocytes (ECs), regulatory-like chondrocytes (RegCs), homeostatic chondrocytes (HomoCs), progenitor chondrocytes (ProCs), hypertrophic chondrocytes (HTCs), pre-hypertrophic chondrocytes (PreHTCs), and fibro-chondrocytes (FCs). Marker expression was visualized using UMAP feature plots and dot plots.

The EC annotation was based on effector chondrocyte-associated markers and published chondrocyte-subpopulation annotations. ECs were analyzed as an annotated chondrocyte state in the integrated cartilage atlas.

**2.5 Oxidative stress gene set and scoring**

A predefined oxidative stress-related gene set was used to evaluate oxidative stress activity at the single-cell level. To improve robustness and reduce dependence on a single algorithm, multiple scoring methods were applied, including AUCell, UCell, singscore, ssGSEA, AddModuleScore, and an integrated scoring strategy.

AUCell evaluates whether a gene set is enriched among highly ranked genes within individual cells. UCell and singscore are rank-based approaches that are relatively robust to differences in sequencing depth. ssGSEA estimates pathway activity for each sample or cell based on gene-set enrichment. AddModuleScore calculates module scores by comparing the average expression of genes in the target gene set with matched control genes. The integrated oxidative stress score was used for major downstream analyses, including comparison among cell types, comparison between normal and OA samples, and HOS cell definition.

**2.6 Identification of high oxidative stress cells**

All cells were ranked according to the integrated oxidative stress score. Cells within the top 10% of oxidative stress scores were defined as high oxidative stress (HOS) cells. The 90th-percentile cutoff was used as a predefined operational threshold to identify cells located in the high-score tail of the oxidative stress distribution. The cell-type composition of HOS cells was calculated to identify cell populations enriched among high oxidative stress cells. The UMAP distribution of HOS cells was compared with the distribution of annotated ECs to assess spatial overlap between HOS cells and EC-enriched regions.

**2.7 CytoTRACE analysis**

CytoTRACE was used to infer the differentiation state and transcriptional potential of HOS cells based on gene expression diversity. Higher CytoTRACE scores were interpreted as reflecting a less differentiated or more transcriptionally active state. CytoTRACE results were used as supplementary evidence describing the transcriptional state of HOS cells rather than as a central mechanistic conclusion.

**2.8 Cell–cell communication analysis**

CellChat was used to infer ligand–receptor-mediated communication among annotated cell populations. A CellChat object was created from the normalized expression matrix and cell identity information. Overexpressed ligands and receptors were identified, and communication probabilities were inferred based on the CellChat ligand–receptor database.

Communication networks were summarized by interaction number and interaction strength. Outgoing and incoming signaling patterns were analyzed to evaluate whether specific cell populations acted primarily as signal senders, receivers, or both. Bubble plots and heatmaps were used to visualize major ligand–receptor signaling pathways. Particular attention was paid to HOS/EC-enriched cells and pathways related to inflammation, extracellular matrix remodeling, oxidative stress, TGF-β, FGF, MIF, SPP1, BMP, EGF, PROS, and other OA-relevant signaling programs. HOS was treated as a score-defined high-oxidative-stress cell group, whereas EC represents the annotated effector chondrocyte subpopulation.

**2.9 High-dimensional weighted gene co-expression network analysis**

High-dimensional weighted gene co-expression network analysis (hdWGCNA) was performed to identify co-expression modules associated with EC-related oxidative stress features. Genes with sufficient variability across cells were selected for network construction. A soft-thresholding power was chosen according to scale-free topology criteria. The adjacency matrix was converted into a topological overlap matrix, and hierarchical clustering was used to identify co-expression modules. Module eigengenes were calculated to summarize module-level expression patterns.

Module eigengenes were correlated with cell-type annotations and oxidative stress scores. Modules associated with the annotated EC population and oxidative stress activity were considered relevant modules. Genes within these modules were retained for functional enrichment analysis and downstream candidate prioritization.

**2.10 Functional enrichment analysis**

Functional enrichment analysis was performed for module-related genes and candidate genes. Gene Ontology (GO) analysis was used to examine biological processes, cellular components, and molecular functions. Kyoto Encyclopedia of Genes and Genomes (KEGG) pathway analysis was used to identify enriched pathways. Disease Ontology (DO) analysis was used to explore disease-related enrichment patterns. Enrichment results were used to provide biological context for candidate genes and modules, with emphasis on oxidative stress, protein homeostasis, inflammatory signaling, energy metabolism, and OA-relevant pathways.

**2.11 Machine learning-based candidate gene prioritization**

To further prioritize candidate genes, five machine learning algorithms were applied: random forest, least absolute shrinkage and selection operator (LASSO) regression, decision tree, gradient boosting machine (GBM), and adaptive best subset selection (ABESS). Random forest was used to evaluate gene importance, and model convergence was assessed using the error curve across increasing numbers of trees. LASSO regression was performed with cross-validation to select genes with non-zero coefficients under the optimal penalty parameter. Decision tree and GBM models were used to identify variables contributing to classification. ABESS was applied to identify an optimal subset of candidate genes.

Genes selected by multiple algorithms were intersected to obtain a robust candidate gene set. Model performance was evaluated using receiver operating characteristic (ROC) curves, area under the curve (AUC) values, decision curve analysis, and confusion matrices. These analyses were used to support candidate selection rather than to propose a clinical diagnostic model.

**2.12 PPI network and hub gene identification**

The overlapping candidate genes identified by machine learning were submitted to STRING to construct a PPI network. Interactions with a combined confidence score greater than 0.4 were retained. The network was visualized using Cytoscape. Network topology was analyzed using Cytoscape tools, and genes with relatively high connectivity were considered potential hub genes. EIF6 and YWHAB were prioritized together with additional hub genes for further evaluation.

**2.13 SMR analysis**

SMR analysis was performed to evaluate whether genetically predicted gene expression was associated with OA risk. OA GWAS summary statistics from GCST005812 were integrated with GTEx v8 whole-blood eQTL data. Cis-eQTLs associated with gene expression were used as instrumental variables. The SMR test was performed to estimate the association between gene expression and OA susceptibility. HEIDI testing was used to distinguish pleiotropic association from linkage. Genes with SMR P < 0.05 and HEIDI P > 0.05 were considered supportive candidates.

Because GTEx whole-blood eQTL data do not directly represent joint tissues or ECs, SMR results were interpreted as supportive genetic evidence for candidate prioritization rather than definitive causal evidence in OA cartilage ECs.

**2.14 Molecular docking**

Molecular docking was performed to explore potential interactions between prioritized candidate proteins. The three-dimensional protein structures of EIF6 and YWHAB were obtained from public structural databases or predicted structural resources. Docking was performed to estimate the potential binding mode and interaction probability between EIF6 and YWHAB. Docking results were used as structural prediction evidence and were not interpreted as direct experimental proof of protein–protein interaction.

**2.15 EC-like model culture and inflammatory stimulation**

C28/I2 human chondrocyte cells were used to establish an inflammatory effector chondrocyte-like model. For consistency with the single-cell annotation, this in vitro model is referred to as an inflammatory EC-like model in the experimental validation sections. Cells were cultured in DMEM high-glucose medium supplemented with 10% fetal bovine serum and 1% penicillin–streptomycin at 37°C in a humidified incubator containing 5% CO₂. Cells at approximately 70–80% confluence were used for experiments.

To induce an inflammatory EC-like state, cells were treated with IL-1β at 10 ng/mL or TNF-α at 10 ng/mL for 24 h. After treatment, cells were collected for qPCR, western blotting, ROS-related fluorescence assays, DHE staining, co-immunoprecipitation, or immunofluorescence according to the experimental design.

**2.16 Validation of the EC-like model**

The identity of the EC-like model was evaluated by qPCR analysis of chondrocyte-associated markers and vascular-lineage markers. Chondrocyte-related markers included SOX9, ACAN, COL2A1, and COMP. Vascular-lineage markers included PECAM1/CD31, CDH5, VWF, and EMCN. Marker expression was assessed by qPCR.

Inflammatory EC-like activation after IL-1β stimulation was evaluated by detecting inflammatory and matrix-remodeling markers, including IL6, CXCL8, PTGS2, and MMP13. Representative EC-associated markers, including KCNMA1, FST, and FGF1, were also assessed to connect the in vitro model with the single-cell EC annotation. The EC-like model was considered supported by chondrocyte-associated marker expression, low vascular-lineage marker expression, inflammatory activation after IL-1β stimulation, and changes in representative EC-associated markers.

**2.17 Transfection and gene manipulation efficiency**

For loss-of-function experiments, EC-like cells were transfected with siRNAs targeting EIF6, YWHAB, or p47phox/NCF1, or with negative control siRNA. For gain-of-function experiments, EC-like cells were transfected with an EIF6 overexpression plasmid or matched empty vector. Transfections were performed using Lipofectamine 3000 according to the manufacturer’s instructions.

The efficiency of EIF6 knockdown, YWHAB knockdown, EIF6 overexpression, and p47phox/NCF1 knockdown was validated by qPCR and/or western blotting before downstream functional analyses. Details of siRNAs, control siRNA, overexpression vector, empty vector, and primer sequences are summarized in Supplementary Table S1.

**2.18 RNA extraction and qPCR**

Total RNA was extracted from treated EC-like cells using an RNA extraction reagent or kit according to the manufacturer’s instructions. RNA concentration and purity were assessed by spectrophotometry. Complementary DNA was synthesized using a reverse transcription kit. qPCR was performed using a SYBR Green-based detection system. Relative gene expression was calculated using the 2−ΔΔCt method, with GAPDH or ACTB as the internal control.

For basal model identity validation, chondrocyte-associated markers and vascular-lineage markers were presented as relative mRNA expression normalized to the internal control. For stimulation and transfection experiments, gene expression was presented as fold change relative to the corresponding control group. Primer sequences are listed in Supplementary Table S1.

**2.19 ROS-related fluorescence detection by DCFH-DA**

Intracellular ROS-related fluorescence was assessed using a DCFH-DA fluorescence probe kit. After experimental treatment, cells were washed with serum-free medium and incubated with DCFH-DA working solution at 37°C in the dark according to the manufacturer’s instructions. Cells were then washed with PBS to remove extracellular probe. Fluorescence images were acquired under identical exposure settings using a fluorescence microscope.

Fluorescence signal was quantified using ImageJ. For each field, fluorescence intensity was measured as integrated density per field and normalized to the control group. At least three independent experiments were performed.

**2.20 DHE staining**

DHE staining was used as an additional ROS-related fluorescence assay. Treated EC-like cells were incubated with DHE working solution under light-protected conditions according to the manufacturer’s instructions. Fluorescence images were acquired under identical exposure settings, and fluorescence intensity was quantified as integrated density per field using ImageJ. DHE staining was used as supportive evidence for ROS-related fluorescence changes and was interpreted together with DCFH-DA staining and p47phox–NOX2 expression.

**2.21 Western blotting**

Total protein was extracted from cultured EC-like cells or mouse joint tissues using RIPA lysis buffer supplemented with protease inhibitors. Protein concentration was determined using a BCA protein assay kit. Equal amounts of protein, typically 20–40 μg per sample, were separated by SDS-PAGE and transferred onto PVDF membranes.

Membranes were blocked with 5% non-fat milk at room temperature for 1 h and incubated with primary antibodies overnight at 4°C. Primary antibodies included anti-EIF6, anti-YWHAB/14-3-3β, anti-p47phox, anti-NOX2, and anti-β-actin. After washing, membranes were incubated with HRP-conjugated secondary antibodies at room temperature. Protein bands were visualized using enhanced chemiluminescence and imaged with a gel imaging system. Band intensity was quantified using ImageJ and normalized to β-actin. Experiments were repeated at least three times.

Original uncropped western blot images were retained and provided as supporting raw data where required.

**2.22 Co-immunoprecipitation**

For co-immunoprecipitation analysis, control and IL-1β-treated EC-like cells were lysed in immunoprecipitation lysis buffer containing protease inhibitors. Equal amounts of protein lysate were incubated with an anti-YWHAB antibody overnight at 4°C. Protein A/G agarose beads were then added and incubated with gentle rotation at 4°C to capture immune complexes.

Beads were washed several times with lysis buffer, and bound proteins were eluted by heating in SDS loading buffer. The precipitated proteins were analyzed by western blotting using antibodies against p47phox and YWHAB. Normal IgG was used as a negative control, and input lysates were included as loading controls. Co-immunoprecipitation results were labeled as “IP: YWHAB; WB: p47phox” and “IP: YWHAB; WB: YWHAB”.

**2.23 Immunofluorescence staining**

EC-like cells were seeded onto glass coverslips and treated according to the experimental design. Cells were fixed with 4% paraformaldehyde, permeabilized with 0.1% Triton X-100, and blocked with bovine serum albumin. Cells were incubated with primary antibodies against the indicated proteins overnight at 4°C. After washing, cells were incubated with fluorescent secondary antibodies at room temperature in the dark. Nuclei were counterstained with DAPI. Fluorescence images were captured using a fluorescence microscope or confocal microscope.

For p65 immunofluorescence, fluorescence intensity was analyzed under matched image acquisition settings. Because p65 staining was used as supportive evidence, the result was interpreted as an NF-κB-related inflammatory signal change rather than definitive quantitative proof of nuclear translocation.

**2.24 DMM-induced OA mouse model**

Specific pathogen-free male C57BL/6J mice aged 10 weeks and weighing 20–25 g were used for animal experiments. Animal experiments were approved by the Laboratory Animal Welfare Ethics Committee of Central People’s Hospital of Zhanjiang under approval number PJ[DW-2025021-01].

Mice were anesthetized with sodium pentobarbital at 50 mg/kg by intraperitoneal injection, and adequate anesthesia was confirmed by the absence of pedal withdrawal reflex. Local 1% lidocaine was applied to the joint surface during surgery. Through a medial parapatellar incision, the joint capsule was exposed, and the medial meniscotibial ligament was transected under a stereomicroscope to establish the destabilization of the medial meniscus (DMM) model. In the Sham group, the joint capsule was exposed without ligament transection. Postoperative analgesia was provided with ibuprofen in drinking water at 30 mg/kg/day for 3 days.

For EIF6 knockdown experiments, mice were assigned to Sham, DMM + siNC, and DMM + siEIF6 groups. For experiments evaluating p47phox-related tissue responses, mice were assigned to DMM + Vector, DMM + EIF6-OE, and DMM + EIF6-OE + si-p47phox groups. In vivo gene manipulation reagents were delivered by intra-articular injection into the operated knee according to the experimental protocol. The dose, vehicle, injection volume, injection frequency, and injection schedule are provided in Supplementary Table S1. Four weeks after surgery, mice were euthanized by gradual-fill CO₂ inhalation at a displacement rate of approximately 30–70% of the chamber volume per minute, followed by cervical dislocation to confirm death. Knee joint tissues were collected for western blotting and immunohistochemistry.

**2.25 Immunohistochemistry**

Mouse knee joint tissues were fixed in 4% paraformaldehyde, decalcified in EDTA, embedded in paraffin, and sectioned at 4 μm thickness. For immunohistochemistry, sections were deparaffinized, rehydrated, and subjected to antigen retrieval. After blocking, sections were incubated with primary antibodies against EIF6, p47phox, or TGF-β overnight at 4°C. Sections were then incubated with HRP-conjugated secondary antibodies. DAB was used for chromogenic development, followed by hematoxylin counterstaining.

Images were captured using a light microscope. Positive staining was quantified using ImageJ. Quantification was performed under consistent threshold settings across groups. IHC staining was also used to validate the efficiency of in vivo EIF6 knockdown, EIF6 overexpression, and p47phox knockdown in the corresponding DMM groups.

**2.26 Statistical analysis**

Statistical analyses were performed using R and/or GraphPad Prism. Quantitative experimental data are presented as mean ± SEM unless otherwise specified. For comparisons between two groups, an unpaired two-tailed Student’s t-test was used when data met parametric assumptions; otherwise, a nonparametric test was applied. For comparisons among three or more groups, one-way analysis of variance followed by Tukey’s or Dunnett’s post hoc test was used as appropriate. Statistical methods for computational analyses were selected according to the corresponding data structure and analysis objective. A P value < 0.05 was considered statistically significant.
